# Benefits of Virtual Reality Balance Training for Patients With Parkinson Disease: Systematic Review, Meta-analysis, and Meta-Regression of a Randomized Controlled Trial

**DOI:** 10.2196/30882

**Published:** 2022-03-01

**Authors:** Jinlong Wu, Hui Zhang, Ziyan Chen, Ruijia Fu, Hao Yang, Hongfa Zeng, Zhanbing Ren

**Affiliations:** 1 Department of Physical Education Shenzhen University Shenzhen China; 2 School of Psychology Shaanxi Normal University Xi'an China

**Keywords:** virtual reality, Parkinson disease, balance, systematic review, meta-analysis, meta-regression, serious games, VR, rehabilitation, VR training

## Abstract

**Background:**

Virtual reality (VR) balance training is increasingly being pursued in biomedical research, specifically with respect to investigating balance ability with VR. However, existing systematic reviews have found inconsistent conclusions about the efficacy of VR in improving balance in Parkinson disease (PD) patients.

**Objective:**

The goal of the research was to evaluate the impact of VR balance training on the balance ability of patients with PD.

**Methods:**

All major databases, including Web of Science, PubMed, Scopus, China National Knowledge Infrastructure, and Wanfang, were searched to identify all relevant studies published in English or Chinese since September 15, 2010. Two researchers independently conducted document retrieval, study selection, data extraction, and methodological quality evaluation.

**Results:**

A total of 16 randomized controlled trials were analyzed (n=583 patients with PD), with the methodological quality evaluation score ranging from 5 to 8 points. A random effects model was selected to combine effect sizes. Meta-analysis showed that the balance ability of PD was significantly improved after VR training compared with the control group (standardized mean difference [SMD] 2.127, 95% CI 1.202 to 3.052, *P*<.001, *I*^2^=95.1, *df*=15). It is worth noting that the intervention platform may be the main reason for heterogeneity. Meta regression analysis showed that no training program could predict the impact of VR training (*P*=.57 to .94) on PD balance ability. Subgroup result showed that a single training time of 0 to 20 minutes (SMD 6.446), 4 to 6 times per week (SMD 4.067), training for 3 to 5 weeks (SMD 62.478), training course reached more than 30 times (SMD 4.405), and 201 to 300 minutes per week (SMD 4.059) maybe have more benefit.

**Conclusions:**

A systematic review and meta-analysis confirmed that VR balance training is a highly effective means to improve balance performance with large effects in PD. In addition, we preliminarily extracted dose-effect relationships for training volume, informing clinicians and practitioners to design effective VR balance training for balance ability. Further research is needed to reveal optimal dose-response relationships following VR balance training.

## Introduction

### Background

Parkinson disease (PD) is the most common neurodegenerative movement disorder and is the result of impaired dopamine-producing nerve cells in the ventral midbrain accompanied by progressive neuronal loss [1,2]. These impairments lead to the maladjustment of motor performance and symptoms such as tremors, stiffness, and reduced limb coordination [3]. Such symptoms can reduce the ability to balance, which often further increases the risk of falling, limits mobility, and reduces the quality of daily life [4-6]. PD has a higher incidence rate among people older than 50 years, with a prevalence rate as high as 4% [4-6]. It is also the fastest growing neurodegenerative condition, impacting 17.5 million individuals globally by 2040. [7,8]. At present, PD is mainly treated by slowing down the course of the disease and improving its core symptoms. The most common clinical scheme for the current treatment of PD is dopamine and deep brain electrical stimulation; however, these have limited and unstable effects in improving posture control and balance [9,10]. In addition, rehabilitation therapy is crucial in the treatment of PD. Exercise has been used as a common rehabilitation therapy, including stretching exercises, strength training, and aerobic exercises to help restore motor function in PD patients [11]. Overall, research shows that rehabilitation therapy based on exercise and cognitive reeducation can effectively improve balance and motor function of PD patients in daily life [12].

As a relatively new intervention measure, virtual reality (VR) technology has become an important auxiliary means in the treatment of various diseases. VR technology involves human-computer interaction technology based on perception (visual, tactile, kinesthetic) and can provide patients with multisensory stimulation and rich virtual scenes, increase the sense of immersion, and realize real-time feedback on physical actions. The main potential mechanisms of VR therapy include the repeatability of virtual tasks, positive feedback from virtual devices, and concrete simulation of a virtual environment. Studies have found that task-oriented repetitive training can enhance the synaptic strength in the brain, continuously affect nerve transmission, and maintain the enhanced functional circuit, thereby accelerating neuroplasticity in patients with neurodegenerative diseases [13]. Therefore, VR technology may be an effective means of treating neurodegenerative diseases such as PD. Under VR conditions, individuals experience multiple sources of sensory stimulation and complete multiple forms of repetitive tasks in a comfortable, safe, and immersive virtual environment, thereby promoting individual functional learning and the transfer of learning function. The potential advantage of VR is that training in VR environments can realize the maintenance and transfer of individual motor skills, which is an important feature of motor skill learning and the basis of real-world behavior [14]. Therefore, VR therapy is considered a supplement to traditional rehabilitation therapy and has been proven to be feasible and effective for treating a variety of neurological diseases [15,16].

At present, optimizing and strengthening the brain compensation mechanism is an important treatment method for PD and other movement disorders [17]. The virtual environment created by VR technology can promote the illusion of bodily movement, increase immersion to enhance the activation of motor brain regions, mobilize the changes of brain neural plasticity, reconstruct the synapses of nervous system cells, and directly train the central nervous system [18,19], resulting in significant benefits to the reorganization and recovery of nerve structure in PD and other neurodegenerative diseases [20]. Existing systematic reviews have found inconsistent conclusions about the efficacy of VR in improving balance in PD patients. One such review found that VR training can effectively improve balance in PD patients compared to other positive interventions [20,21]; however, other studies did not find these effects [22], a discrepancy that may be due to publication bias and diversity of interventions [20]. In addition, sample sizes of randomized controlled trials (RCTs) are currently insufficient to explore the dose effect of VR technology training on improving PD balance [20].

### Objectives

In view of the current state of research consensus, the primary objective of this study was to review and analyze the existing RCT studies to verify whether VR training can improve balance in PD patients. Positive findings would prompt further investigation of an optimal dose of VR training to improve the balance of PD patients with the eventual goal of providing clinical workers with stronger theoretical support for VR training in the treatment of PD.

## Methods

This study was conducted in accordance with the guidelines of Preferred Reporting Items for Systematic Reviews and Meta-analyses [20].

### Research Retrieval

We searched 5 databases: Web of Science, PubMed, Scopus, China National Knowledge Infrastructure, and Wanfang. The last retrieval date was September 15, 2020. We conducted a literature retrieval using three sets of keywords: (1) virtual reality, VR, Kinect, Wii, Xbox; (2) Parkinson, parkinsonian disorders, Parkinson, parkinsonism, Parkinson disease, PD; and (3) balance, equilibrium, dynamic postural control; the three groups of keywords were retrieved using the AND combination in the database. The Chinese database used the Chinese translation of the above keywords. Finally, the references of all included studies and relevant systematic reviews [20,21,23-27] were manually searched to further identify relevant studies.

### Inclusion Criteria

#### Research Type

The studies were either RCTs or nonrandomized controlled trials, where nonrandomized trials generated by pseudorandom or nonrandom sequences were defined as nonrandomized controlled trials. Those that did not involve a comparison group or did not report intergroup comparisons were excluded. In the case of cross-sectional designs, a set of postmortem and qualitative analyses were also excluded. We further excluded reviews, conference summaries, and book chapters and restricted our research to articles written in the English or Chinese language.

#### Participant Type

The subjects of the study were PD patients aged 18 years or older who had been formally diagnosed by the hospital or by internationally recognized diagnostic criteria. There were no restrictions on sex, course of disease, or severity of the disease.

#### Intervention Type

The VR training immersion included a variety of modes, such as nonimmersive, semi-immersive, and fully immersive. The control group consisted of a wait group, routine physical therapy, or other types of treatment such as drug therapy.

#### Types of Outcome Indicators

In order to improve the quality of research, it was necessary to use effective and reliable tools to measure balance ability. Due to the high task specificity of balance ability, we selected the Berg Balance Scale (BBS), which is widely used to assess overall balance ability of patients with movement disorders [28]. The full assessment includes 14 balance-related activities, in which higher scores indicate better balance ability. The maximum possible score is 56 points, and a score below 40 indicates a risk of declining balance ability.

#### Study Selection and Data Extraction

We conducted independent screening of studies based on the title, abstract, and full text, and two researchers discussed the results before reaching a consensus. In the event of no consensus, a third researcher made the final decision.

Descriptive data were extracted after reading the full text. The extracted content comprised 3 categories: literature characteristics, participant characteristics, and intervention plan. Literature characteristics included first author, number of years of publication, country, and language. Participant characteristics included diagnostic criteria (diagnostic tools), number of participants (number and sex ratio of the experimental group and control group), and age. In order to determine the dose-response relationship of VR training to improve balance in PD patients, the balance training scheme was coded as follows: training group (experimental group and control group), single training duration, training frequency, total number of training sessions, weekly training duration and total training duration, and VR training platform [27,29]. We extracted the quantitative balance data in the experimental and control group in each eligible RCT study (ie, BBS scores before and after intervention were extracted), and the results must be quantitative data that can be used for effect size calculation. If there were multiple control groups in the study, only the control group with active intervention measures was extracted.

### Quality Assessment

We used the Physiotherapy Evidence Database (PEDro) Scale to assess the methodological quality of clinical trials in physiotherapy and rehabilitation [30]. The quality evaluation was carried out using the PEDro quality assessment sheet to evaluate the treatment included in the study. The evaluation criteria were as follows: eligibility criteria, randomization, concealed allocation, baseline equivalence, blinding of participants, blinding of instructors, blinding of assessors, retention rate of 85%, missing data management (intent-to-treat analysis), between-group analysis, and measures of variability. One point was awarded if the information was explicitly presented, with a maximum of 9 points per study. If the above information was clear in the study, 1 point was awarded; if not, 0 points were awarded, while the maximum score for each study was 11 points. According to the scores, the quality of these studies can be divided into 4 grades: excellent (>9 points), good (6 to 8 points), fair (4 to 5 points), and poor (<4 points) quality. For studies that did not provide enough information to complete the assessment, the authors requested relevant information via email; a lack of author response resulted in a designation of ambiguous information.

### Data Analysis

In order to explore the benefits of VR training on PD balance, we combined extracted BBS scale data with effect size using the statistical software STATA (version 15.1, StataCorp LLC), resulting in a random effect model for further analysis calculation. The standardized mean difference (SMD) was selected as the index of effect scale for statistics. The effect size indicated the degree of impact of VR training on PD balance ability, where SMD <0.20 indicated a negligible effect, 0.20 to <0.50 indicated a small effect, 0.50 to <0.80 indicated a large effect, and an SMD >0.80 indicated a larger effect [31].

We used the statistic *I*^2^ to evaluate the heterogeneity of the selected studies, in which a larger *I*^2^ statistic indicates greater heterogeneity [31]. In addition, we used funnel plots and Egger tests to evaluate publication bias [32]. Subgroup analyses were used to estimate the impact of publication bias on the interpretation of results [33]. We further used meta-regression models to analyze the effect of the VR training program according to length and frequency of training courses, total time of weekly training courses, total number, and time of training courses. Therefore, this meta-regression part was not analyzed in this study due to its failure in quantifying the intensity of treatment. For studies that did not provide the above data, the author was contacted by email to obtain the data, so as to improve the quality of this research.

## Results

### Study Selection

We retrieved a total of 491 records through electronic databases: 200 records were retrieved by Web of Science, 84 records were retrieved by PubMed, 144 records were retrieved by Scopus, 53 records were retrieved by China National Knowledge Infrastructure, and 10 records were retrieved by Wanfang. In addition, 10 records were added manually.

**Figure 1:**
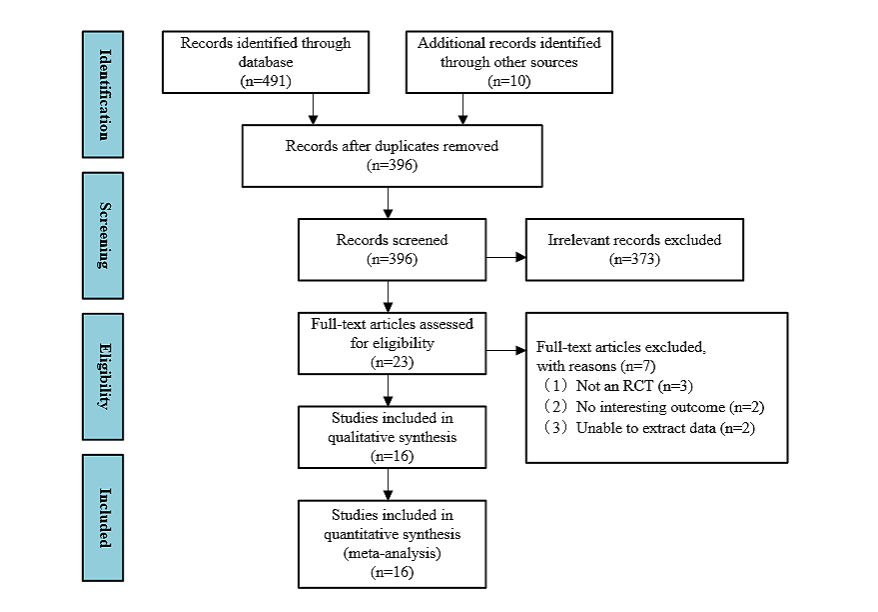
The flow of literature search and study selection. RCT: randomized controlled trial.

We then excluded 105 duplicate records. The remaining 393 records were further screened by titles and abstracts, and 373 records that did not meet the study criteria were deleted. We then evaluated 23 studies for full-text qualification, and 7 studies were found not to meet the predetermined inclusion criteria. Finally, a total of 16 studies were identified for meta-analysis. The flow chart of literature retrieval and study selection is shown in Figure 1.

### Research Characteristics

Table 1 lists the characteristics and descriptions of the 16 studies published from 2012 to 2020, involving 583 PD (experimental group: 291; control group: 292) participants aged 55 to 75 years in the included study. All the included PDs were tested using diagnostic tools such as the Movement Disorder Society Clinical Diagnostic Criteria for Parkinson disease (MDS-PD), the United Kingdom Parkinson Disease Society Brain Bank criteria (UK-PDSBB), and the Chinese Parkinson Diagnostic Criteria, or patients who had been clinically diagnosed as PD by doctors, of which six [33] studies were conducted in China, two [34,35] studies were conducted in the United Kingdom, two [34,35] studies were conducted in Brazil, and two [36,37] studies were conducted in Taiwan, including one study each in South Korea [38], Italy [39], the United States [40], and Istanbul [41]. All of the included studies were comparative studies between the VR group and conventional rehabilitation.

The VR training program has a single duration between 30 and 60 minutes, with frequency varying from 2 to 5 times per week and duration ranging from 4 to 12 weeks. All of the 16 studies were nonimmersive VR devices, including Xbox (Microsoft) and Wii Fit (Nintendo Co Ltd). Among them, four studies [35,36,41,42] used Xbox Kinect equipment and five studies [38-40,43,44] used Wii Fit equipment. Additionally, two studies [45,46] used Silverfit 3D motion capture analysis system, three studies [37,47,48] were personalized development VR rehabilitation systems, and one study [34] only reported device components and game types, among which one study [49] did not report the name of VR device. The outcome indicators of interest in this study were all BBG scales, thereby having no separate description in the study characteristics.

**Table 1:** Study characteristics.

Reference				Participant characteristics				Intervention protocol		
Authors	Country, Language	Diagnostic criteria	Hoehn-Yahr classification		N, male/female	Age	Method		Session duration/frequency/period/# sessions/duration of VR^a^ treatment per week	Platform
Chen et al [33]	China, Chinese	Chinese Parkinson Diagnostic Criteria	EG^b^: 2.52 (SD 0.51); CG^c^: 2.57 (SD 0.50)		EG: 23, 14/9; CG: 23, 12/11	EG: 62.09 (SD 6.11); CG: 64.65 (SD 5.06)	EG: VR training; CG: routine rehabilitation training		50 min/5 times per wk/6 wk/30/250 min	BioFlex-FP posture control evaluation and training system
Cheng et al [47]	China, Chinese	MDS-PD^d^	EG: 3.1 (SD 0.6); CG: 3.0 (SD 0.6)		EG: 20, 9/11; CG: 20, 8/12	EG: 59.2 (SD 7.3); CG: 58.6 (SD 7.5)	EG: VR training+ routine rehabilitation training (40 min); CG: routine rehabilitation training		20 min/2 times per wk/8 wk/16/40 min	Silverfit 3D motion capture analysis system
Sun et al [46]	China, Chinese	Clinical diagnosis	Total: 1-4		EG: 30, 19/11; CG: 30, 21/9	EG: 61.43 (SD 7.34); CG: 62.54 (SD 6.98)	EG: VR training + strengthen muscle strength training (20 min); CG: routine rehabilitation training + medication		20 min/5 times per wk/4 wk/20/100 min	Silverfit 3D motion capture analysis system
Lin et al [48]	China, Chinese	Chinese Parkinson Diagnostic Criteria	Total: 2.5-4		EG: 18, 12/6; CG: 15, 10/5	EG: 61.4 (SD 8.2); CG: 62.1 (SD 6.3)	EG: VR training + medication; CG: routine rehabilitation training + medication		30 min/5 times per wk/4 wk/20/150 min	Xbox (Microsoft Corp)
Liu et al [45]	China, Chinese	Chinese Parkinson diagnostic criteria	Total: 3		EG: 21, 11/10; CG: 21, 12/9	EG: 60.9 (SD 7.20); CG: 63.9 (SD 5.82)	EG: VR training; CG: routine rehabilitation training		20 min/5 times per wk/4 wk/20/150 min	Computer-assisted rehabilitation environment (CAREN)
Pompeu et al [35]	Brazil, English	Clinical diagnosis	Total: 1-2		Total: 32, 17/15	Total: 67.4 (SD 8.1)	EG: VR training; CG: routine rehabilitation training		30 min/2 times per wk/7 wk/14/60 min	Wii Fit (Nintendo Co Ltd)
Van et al [34]	UK, English	UK-PDSBB^e^	Total: 2-3		EG: 17, 12/5; CG: 16, 8/8	EG: 66.3 (SD 6.39); CG: 68.8 (SD 9.68)	EG: VR training; CG: routine rehabilitation training		60 min/2 times per wk/5 wk/10/120 min	Workstations consisted of a flat-panel LCD monitor connected to a PC containing a total of 6 commercially available, interactive dynamic balance exercises (Motek Medical)
Lee et al [37]	Korea, English	Clinical diagnosis	NR^f^		EG: 10, 5/5; CG: 10, 5/5	EG: 68.4 (SD 2.9); CG: 70.1 (SD 3.3)	EG: VR training + neurodevelopmental therapy (NR) + functional electrical stimulation therapy (NR); CG: routine rehabilitation training		30 min/5 times per wk/6 wk/30/150 min	k-pop dance festival (Nintendo Inc) game for the Wii (Nintendo Inc) video game system
Shih et al [41]	Taiwan, English	UK-PDSBB	EG: 1.6 (SD 0.84) ; CG: 1.4 (SD 0.52)		EG: 10, 9/1; CG: 10, 7/3	EG: 67.5 (SD 9.96); CG: 68.8 (SD 9.67)	EG: VR training; CG: routine rehabilitation training		50 min/2 times per wk/8 wk/16/100 min	Kinect sensor (Microsoft Corp)
Yang et al [36]	Taiwan, English	UK-PDSBB	Total: 2-3		EG: 11, 7/4; CG: 12, 7/5	EG: 75.4 (SD 6.3); CG: 72.5 (SD 8.4)	EG: VR training; CG: routine rehabilitation training		50 min/2 times per wk/6 wk/12/100 min	VR balance training system included a 22-inch all-in-one touchscreen computer (Micro-Star International Co Ltd) and a wireless balance board
Ozgonenel et al [40]	Istanbul, English	Clinical diagnosis	Total: 1-3		EG: 15, 10/5; CG: 18, 12/6	EG: 64; CG: 65	EG: VR training + routine rehabilitation training (NR); CG: balance rehabilitation training		20 min/3 times per wk/5 wk/15/60 min	Xbox (Microsoft Corp)
Gandolfi et al [38]	Italy, English	UK-PDSBB	Total: 1-3		EG: 38, 23/15; CG: 38, 28/10	EG: 67.45 (SD 7.18); CG: 69.84 (SD 9.41)	EG: VR training + balance rehabilitation training (NR); CG: sensory integration balance rehabilitation training		50 min/3 times per wk/7 wk/21/150 min	Wii Fit gaming system and balance board (Nintendo Co Ltd)
Ribas et al [43]	Brazil, English	UK-PDSBB	Total: 2-3		EG: 10, 4/6; CG: 10, 4/6	EG: 61.7. (SD 6.83); CG: 60.20 (SD 11.29)	EG: VR training; CG: routine rehabilitation training		30 min/2 times per wk/12 wk/24/60 min	Wii Fit gaming system, and balance board (Nintendo Co Ltd)
Santos et al [39]	US, English	UK-PDSBB	EG: 1.4 (SD 0.6); CG: 1.3 (SD 0.3)		EG: 13; CG: 14	EG: 61.7 (SD 7.3); CG: 64.5 (SD 9.8)	EG: VR training; CG: routine rehabilitation training		50 min/2 times per wk/8 wk/16/100 min	Wii Fit gaming system and balance board (Nintendo Co Ltd)
Feng et al [42]	China, English	UK-PDSBB	EG: 3.03 (SD 0.55); CG: 2.97 (SD 2.66)		EG: 14, 8/7; CG: 14, 9/6	EG: 67.47 (SD 4.79); CG: 66.93 (SD 4.64)	EG: VR training + medication; CG: routine rehabilitation training + medication		45 min/5 times per wk/12 wk/60/225 min	NR
Tollár et al [49]	UK, English	UK-PDSBB	EG: 2.3 (SD 0.48); CG: 2.4 (SD 0.51)		EG: 25, 12/13; CG: 25, 11/14	EG: 70.0 (SD 4.69); CG: 70.6 (SD 4.10)	EG: VR training + medication; CG: routine rehabilitation training + medication		60 min/5 times per wk/5 wk/25/300 min	EXE^g^ used the following 3 visual feedback modules of the Xbox 360 core system (Kinect Adventures, Microsoft Corp)

^a^VR: virtual reality.

^b^EG: experiment group.

^c^CG: control group.

^d^MDS-PD: Movement Disorder Society clinical diagnostic criteria for Parkinson disease.

^e^UK-PDSBB: United Kingdom Parkinson Disease Society Brain Bank criteria.

^f^NR: no report.

^g^EXE: exergame.

### Methodological Quality

The results of methodological assessment are shown in Table 2, with scores ranging from 5 to 8 and an average score of 6.56. Specifically, the most common methodological flaw in these studies was that there was no allocation concealment. Due to the difficulty in conducting blind training in VR training and routine rehabilitation training, most of the included studies did not implement subject blindness, therapist blindness, or evaluator blindness.

**Table 2:** Study quality assessment of eligible studies.

Author	Eligibility criteria	Randomization	Concealed allocation	Similar baseline	Blinding of participants	Blinding of instructors	Blinding of assessors	Retention >85%	Intent-to-treat analysis	Between-group comparison	Point measure and measures of variability	Sum score
Chen et al [33]	1^a^	1	0^b^	1	0	0	0	1	0	1	1	6
Cheng et al [47]	1	1	0	1	0	0	0	0	0	1	1	5
Sun et al [46]	1	1	0	1	0	0	0	1	0	1	1	6
Lin et al [48]	1	1	0	1	0	0	1	1	0	1	1	7
Liu et al [45]	1	1	0	1	0	0	0	1	0	1	1	6
Pompeu et al [35]	1	1	0	1	0	0	1	1	0	1	1	7
Van et al [34]	1	1	1	1	0	0	1	0	0	1	1	7
Lee et al [37]	1	1	0	0	0	0	1	0	0	1	1	5
Shih et al [41]	1	1	1	1	1	0	0	1	0	1	1	8
Yang et al [36]	1	1	1	0	0	0	1	1	1	1	1	8
Ozgonenel et al [40]	1	1	0	1	1	0	0	0	0	1	1	6
Gandolfi et al [38]	1	1	0	1	0	0	1	1	0	1	1	7
Ribas et al [43]	1	1	0	1	0	0	0	1	0	1	1	6
Santos et al [39]	1	1	1	1	0	0	0	1	0	1	1	7
Feng et al [42]	1	1	0	1	0	0	0	1	0	1	1	6
Tollár et al [49]	1	1	1	1	1	0	0	1	0	1	1	8

^a^Explicitly described and present in details.

^b^Absent, inadequately described, or unclear.

### Effect Of VR Training on Improving PD Balance Ability

**Figure 2:**
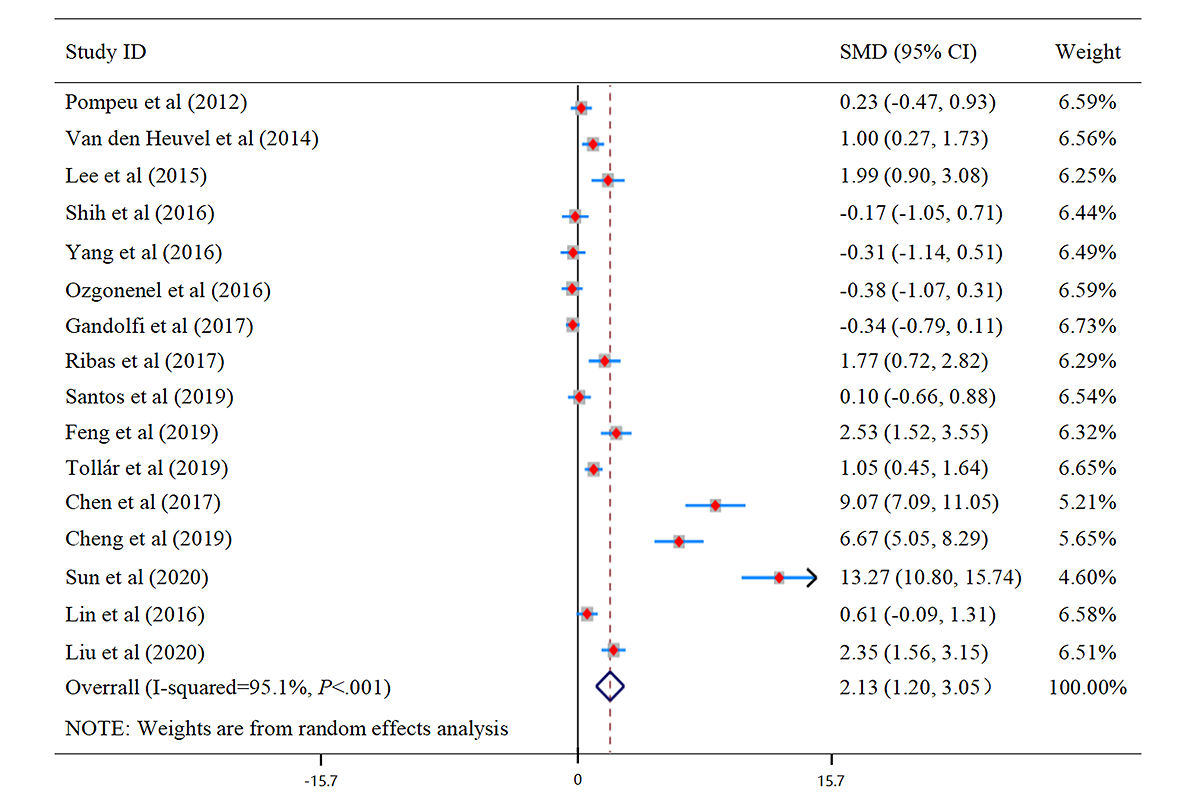
Forest plot for balance. SMD: standardized mean difference.

**Figure 3:**
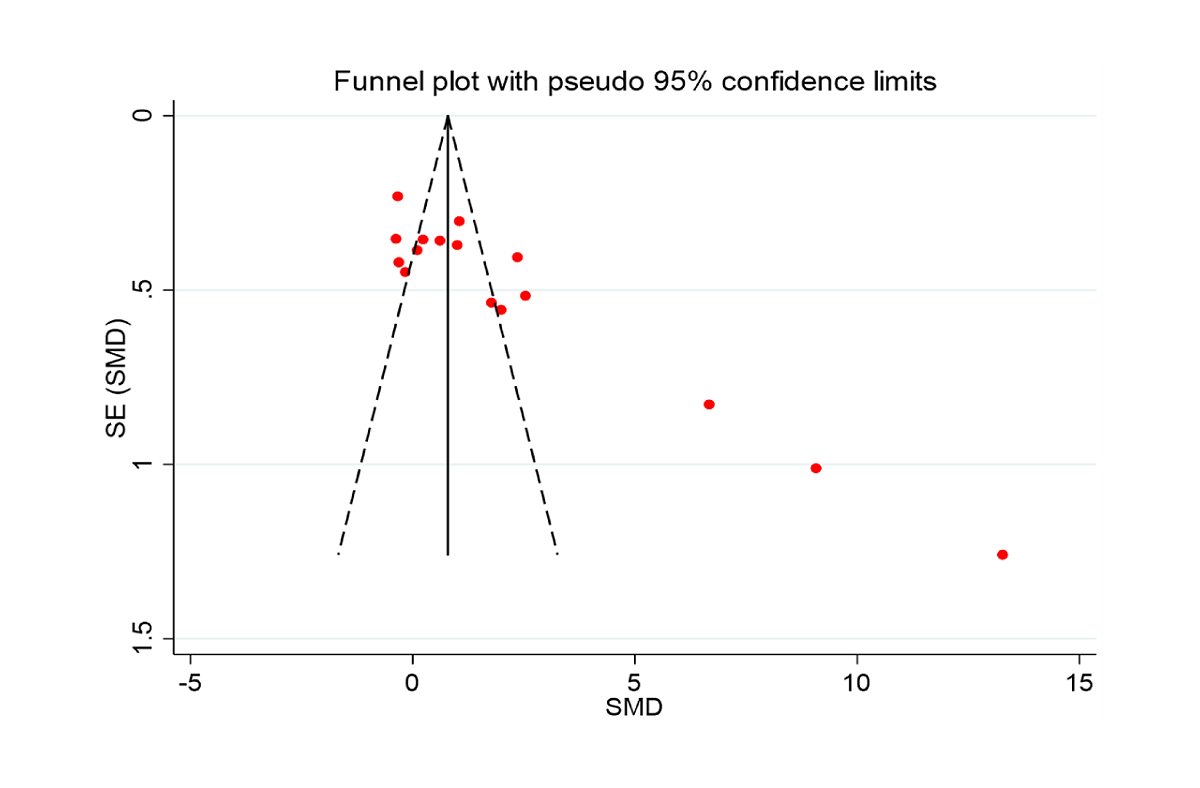
Funnel plot for balance. SMD: standardized mean difference.

A total of 16 studies investigated the effects of VR training on improving PD balance ability. These data were analyzed for random effects to combine the results. Figures 2 and 3 illustrate the effects of VR balance training on balance. The meta-analysis results showed that SMD 2.13, 95% CI 1.20 to 3.05, *P*<.001 (see Figure 2). This indicates the balance ability of PD was significantly improved after VR training compared with the control group. The Egger test results found no significant publication bias (Egger regression intercept 10.69691, *P*<.001, *df*=15). However, results of funnel plots showed that three studies [49] exhibited asymmetry. That is, the included studies may have publication bias (see Figure 3). It is worth noting that the interstudy heterogeneity was very high, the value of *I*^2^ with over 75% (*I*^2^=95.1, *P*<.001).

### Moderating Variable Analysis: Impact of Premode and Intervention Platform

We conducted subgroup analysis on intervention modes and intervention platforms (excluding one study [49] that did not report VR equipment) to clarify the regulatory effect of these 2 variables on PD balance ability. Subgroup analysis showed that training methods had great influence on balance ability. It is worth noting that intervention platform may be the main reason for heterogeneity, as subgroup analysis of the VR rehabilitation and commercial game VR platforms found that the heterogeneity of VR rehabilitation platforms (*I*^2^=96.8%) was very large, while that of commercial game VR reality platforms was relatively small; however, there was no significant difference in the improvement of PD balance ability of commercial game VR platforms (SMD 0.447, 95% CI 0.03 to 0.898), which may be the reason for the different conclusions in the previous review. Nevertheless, due to the lack of research on VR rehabilitation platforms, this result should be treated with caution (Table 3).

**Table 3:** Results of the overall balance subgroup analyses on the influence of intervention method and intervention platform on virtual reality training in Parkinson disease.

Independent training modality		SMD^a^	95% CI	*z* value (*P* value)	*I*^2b^ (%)	*df*	*P* value between groups
**Intervention method**							
	Virtual reality mixed physical rehabilitation methods	2.64	0.699 to 4.588	2.66	96.8	5	<.001
	Virtual reality	1.83	0.795 to 2.872	3.46	93.5	9	<.001
**Intervention platform**							
	Virtual rehabilitation platform	7.75	3.134 to 12.365	3.29	97.1	3	<.001
	Commercial gaming virtual reality platform	0.45	–0.0.3 to 0.898	1.95	76.2	10	<.001

^a^SMD: standardized mean difference.

^b^*I*^2^: heterogeneity between studies.

### Impact of VR Balance Training and the Dose-Effect Relationship

In order to explore the optimal training variables, this study first conducted a meta-regression to explore the effect of a training program on the effectiveness of PD balance. In addition to meta-regression (Table 3), subgroup analysis was conducted to preliminarily analyze the effect size of each training program subgroup and presented for the preliminary analysis of dose response relationships (Table 4).

**Table 4:** Results for the subgroup analyses on the effects of different categories of respective training volume on balance ability.

Training volume	Coefficient	SE	95% CI	*z* value	2-sided *P* value
Training period	–0.0558	0.2075	–0.4626 to 0.3509	–0.27	.79
Training frequency	1.9711	3.4458	–4.7826 to 8.7248	0.57	.57
Number of sessions	0.4613	1.3035	–2.0935 to 3.0162	0.35	.72
Single session duration	–0.1145	0.3182	–0.7381 to 0.5091	0.36	.72
Total duration per week	–2.1933	0.0572	–0.1167 to 0.1077	–0.08	.94

### Meta-Regression Analysis of Volume of VR Balance Training

Due to the low number of studies, we performed meta-regression only for the subcategory training volume. The regression analysis revealed that no variable within the training volume subcategory (single training duration, training frequency, total number of training sessions, weekly training duration, total training duration) produced significant effects (*P*=.57 to .94) on the ability of improving balance level. That is, in the included studies, we could not use time or number of training sessions to predict the ability of improving balance level (see Table 4).

### Dose-Effect Relationship of VR Balance Ability

#### Single Training Time

A total of 13 studies were included in this subanalysis, and the time range of single training from 0 to 20 minutes produced large effects on balance, with SMD of 6.446 (95% CI –1.098 to 13.990; *z*=1.67; *I*^2^=98%; *df*=2; see Table 5).

**Table 5:** Results for the subgroup analyses on the effects of different categories of respective training volume on overall balance.

Independent training modality			SMD^a^		95% CI		*z* value (*P* value)		*I*^2^^b^ (%)		*df*		*P* value between groups
**Single training time**													
	0-20 min	6.446		–1.098 to 13.990		1.67 (.094)		98.7		2		<.001	
	21-40 min	1.050		0.122 to 1.979		2.22 (.027)		89.4		5		<.001	
	41-60 min	1.644		0.378 to 2.910		2.54 (.011)		93.6		6		<.001	
**Training frequency**													
	1-3 sessions per week	0.770		–0.060 to 1.600		1.82 (.069)		90.6		8		<.001	
	4-6 sessions per week	4.067		2.223 to 5.911		4.32 (<.001)		96.1		6		<.001	
**Training period**													
	3-5 weeks	2.478		0.893 to 4.063		3.06 (.002)		95.9		5		<.001	
	6-8 weeks	1.917		0.515 to 3.320		2.68 (.007)		95.5		7		<.001	
	9-12 weeks	2.166		1.417 to 2.914		5.67 (.001)		5.30		1		<.001	
**Total number of training sessions**													
	10-20 sessions	0.844		–0.210 to 1.898		1.57 (.117)		91.4		6		<.001	
	21-30 sessions	2.638		1.003 to 4.272		3.16 (.002)		96.5		5		<.001	
	＞30 sessions	4.405		1.102 to 7.707		2.61 (.009)		95.0		2		<.001	
**Total duration of VR^c^ training per week**													
	0-100 min	2.313		0.671 to 3.955		2.76 (.006)		96.1		7		<.001	
	101-200 min	1.079		0.046 to 2.112		2.05 (.041)		90.6		4		<.001	
	201-300 min	4.059		0.738 to 7.379		2.40 (.017)		96.7		2		<.001	

^a^SMD: standardized mean difference.

^b^*I^2^*: heterogeneity between studies.

^c^VR: virtual reality.

#### Training Frequency

The training frequency was 1 to 6 sessions per week in the 14 studies, and the result showed the SMD for 4 to 6 sessions per week was 4.067 (95% CI 2.223 to 5.911; *z*=4.32 ; *I*^2^=96.1%; *df*=7), indicative of large effects (see Table 5).

#### Training Period

In 13 studies, during the training period, a time under training of 3 to 5 weeks with SMD of 2.478 appears most effective compared other training period (95% CI 0.893 to 4.063; *z*=3.03; *I*^2^=95.9%; *df*=5) in this subanalysis (see Table 5).

#### Total Number of Training Sessions

Based on data from 13 studies, we computed the effect of total number of training sessions (range: 10 to 20 sessions, 21 to 30 sessions, ＞30 sessions) of VR training for balance ability. Above 30 sessions in total, with SMD of 4.405 is the most effective for balance ability (95% CI 1.102 to 7.707; *z*=2.61; *I*^2^=95.0%; *df*=2; see Table 5).

#### Total Duration of VR Training per Week

In 14 studies, we also computed the effect of total duration of VR training per week (range 0 to 100 minutes, 101 to 200 minutes, 201 to 300 minutes). The largest effect was shown for 201 to 300 minutes of training per week (SMD 4.059; 95% CI 0.738 to 7.379; *z*=2.40; *I*^2^=96.7%; *df*=2; see Table 5).

## Discussion

### Summary

In this study, we examined the effect of VR technology on improving PD balance ability. Data analysis of 16 RCTs showed that (1) VR technology has a significant impact on the improvement of PD balance ability, (2) different VR platforms may have different effects on PD balance and the effect of rehabilitation platforms is superior to commercial platforms, and (3) variability in training methods cannot predict the effect of VR training on PD balance ability.

### Impact of VR on PD Balance Ability

VR can provide visual and auditory feedback to PD patients, thereby minimizing the lack of movement caused by internal prompts due to reduced dopamine consumption [49]. In addition, the real-time feedback provided by VR platforms may also facilitate the movement process [50]. PD, by contrast, shows insufficient interaction between vestibular and proprioceptive systems, leading to changes in human biomechanics that affect balance. The visual and auditory input provided by VR can therefore effectively integrate the feedback of vestibular receptors and proprioceptive receptors in PD patients, improving PD balance ability [51]. VR training has been shown to stimulate the integration of PD-related cognitive functions such as attention and executive ability, as well as stimulate the reward mechanism of the brain. Moreover, success in alleviating PD symptoms using VR training requires attention, sensory integration, and treatment of stimuli in VR environments and may be more effective than traditional rehabilitation programs [52,53]. A functional near-infrared spectroscopy study found that VR training increases the blood oxygen concentration in the prefrontal cortex of the brain, indicating that prefrontal cortex is involved in VR balance task and degree of activation is modulated by VR task difficulty [54].

Long-term physical therapy is also beneficial for PD. Due to the incentive stimulation of VR technology, it can maintain motivation during the long-term rehabilitation process and improve compliance so as to reduce the negative impacts during the rehabilitation process [55]. In addition, studies have indicated that VR balance training and traditional interventions can increase the muscle strength and balance ability of patients with anterior cruciate ligament injury [56], as well as improve coordination and reflex conduction [57]. However, due to the fact that the measurement methods are not often very precise, it is difficult to find optimal balance points between the two.

### Moderating Effect

Our subgroup analyses of intervention methods and intervention platforms found no statistically significant difference between intervention methods, nor did heterogeneity decrease significantly. However, we found VR rehabilitation platforms to be significantly heterogeneous, and the commercial game VR platform’s *I*^2^ reduced to 71.2%, which may be the main source of heterogeneity in this study. It should also be noted that we found no statistically significant effect of VR platform training of commercial games; that is, the effects of VR training and traditional rehabilitation training on PD balance ability were similar, which is consistent with previous research results [22]. We therefore conclude that the VR platform itself may be one of the reasons for the inconsistency of previous results, although a small sample size means that this result should also be treated with caution.

Although commercial systems such as Wii are now included in the neurorehabilitation program [58], such commercial games are not personalized and do not allow precise interactions between individuals and VR environments [58]. Furthermore, the overall safety and clinical nature of sports-based computer games have not been fully confirmed [52]. In addition, VR device design may not be optimized for PD, which may damage the experience and safety of PD patients [52].

### Impact of VR Training and the Dose-Effect Relationship

As discussed previously, VR balance training is of great benefit to PD patients. Our results showed that no particular training method could predict the effect of VR balance training on PD balance performance. With the exception of meta-regression, subgroup analysis was conducted for each training program to explore the magnitude of the effect of a specific subgroup.

In addition to meta-regression, subgroup analysis was conducted for each training program to explore the magnitude of the effect of a specific subgroup and explain the effect of the amount of certain subgroups. The most successful VR training program to enhance PD balance was a single training duration of 0 to 20 minutes, 4 to 6 times a week, training for 3 to 5 weeks, training course reached more than 30 times, 201 to 300 minutes per week. However, this study only provides a preliminary analysis of the dose-response relationship through subgroup analysis and should be more widely explored in future research.

### Limitations

The biggest limitation of this study is that there was considerable heterogeneity across studies (ie, *I*^2^=95.1%). Even if subgroup analysis was performed to observe the underlying causes of heterogeneity, the results should be treated with caution. Another limitation is that due to the limited number of available studies, there were data from only 16 RCTs, and the intervention plans included in the study varied widely, making it difficult for subgroup analyses to provide a clear picture of the dose-response relationship. Moreover, due to the fact that the BBS scale is assessed manually, there may be a degree of error in the balance ability data.

### Conclusion

The systematic review and meta-analysis confirmed that VR balance training is a highly effective means to improve balance performance with large effects in PD. In addition, we preliminarily extracted dose-effect relationships for training volume, informing clinicians and practitioners to design effective VR balance training for balance ability. Future studies should particularly focus on the detailed description of training variables, so as to further analyze the dose-effect relationship of VR balance training in PD.

## Abbreviations

BBS: Berg Balance Scale
MDS-PD: Movement Disorder Society Clinical Diagnostic Criteria for Parkinson disease
PD: Parkinson disease
PEDro scale: Physiotherapy Evidence Database
RCT: randomized controlled trial
SMD: standardized mean difference
UK-PDSBB: United Kingdom Parkinson Disease Society Brain Bank
VR: virtual reality
